# Characterization of a novel amidohydrolase with promiscuous esterase activity from a soil metagenomic library and its application in degradation of amide herbicides

**DOI:** 10.1007/s11356-024-32362-6

**Published:** 2024-02-21

**Authors:** Shengwei Sun, Wanqi Chen, Kailin Peng, Xueyingzi Chen, Jinju Chen

**Affiliations:** 1 Key Laboratory of Food Processing and Quality Control, College of Food Science and Technology, Nanjing Agricultural University, Nanjing, 210095 People’s Republic of China; 2 School of Engineering, Newcastle University, Newcastle Upon Tyne, NE1 7RU UK; 3 Department of Materials, Loughborough University, Loughborough, LE11 3TU UK

**Keywords:** Amide herbicide, Environmental pollution, Amidohydrolase, DET, Metagenomic library, Molecular docking

## Abstract

Amide herbicides have been extensively used worldwide and have received substantial attention due to their adverse environmental effects. Here, a novel amidohydrolase gene was identified from a soil metagenomic library using diethyl terephthalate (DET) as a screening substrate. The recombinant enzyme, AmiH52, was heterologously expressed in *Escherichia coli* and later purified and characterized, with the highest activity occurring at 40 ℃ and pH 8.0. AmiH52 was demonstrated to have both esterase and amidohydrolase activities, which exhibited highly specific activity for *p*-nitrophenyl butyrate (2669 U/mg) and degrading activity against several amide herbicides. In particular, it displayed the strongest activity against propanil, with a high degradation rate of 84% at 8 h. A GC–MS analysis revealed that propanil was transformed into 3,4-dichloroaniline (3,4-DCA) during this degradation. The molecular interactions and binding stability were then analyzed by molecular docking and molecular dynamics simulation, which revealed that several key amino acid residues, including Tyr164, Trp66, Ala59, Val283, Arg58, His33, His191, and His226, are involved in the specific interactions with propanil. This study provides a function-driven screening method for amide herbicide hydrolase from the metagenomic libraries and a promising propanil-degrading enzyme (AmiH52) for potential applications in environmental remediation.

**Supplementary Information:**

The online version contains supplementary material available at 10.1007/s11356-024-32362-6.

## Introduction

With the continuous refinement and development of agriculture, the global consumption of pesticides has grown significantly from 1990 to 2020 (Statista, 2022). Among these various pesticides, amide herbicides have been used extensively to increase crop yield and farm labor efficiency (Ni et al. 2023; Qi et al. 2020), accounting for the second-largest proportion of herbicides used in agriculture (Wang et al. 2023). Moreover, in 2018 alone, global amide herbicide usage was more than 170,000 t (Ni et al. 2023). Due to this widespread use, mobility, and persistence, amide herbicides have been frequently detected in the soil environment. For example, a range of 0.03–709.37 mg/kg acetochlor was detected in the riparian soils and sediments of northeastern China, and its residual concentration was 54.76 mg/kg for maize land (Li et al. 2018). Furthermore, with the overdose and irrational use of toxic herbicides exceeding the maximum soil retention capacity, the residues diffuse to the surface waters and groundwater, posing a serious threat to food safety and public health (Tan et al. 2020). It is, therefore, necessary to take effective measures to reduce herbicide residue pollution.

Biodegradation has been recognized as the most effective intervention for removing amide herbicide residues in the environment (Huang et al. 2017). Research has suggested that the amide herbicides in the soil can be degraded through microbial catabolism, which is a more efficient degradation method than hydrolysis and photolysis (Sur and Sathiavelu 2022). As a result, many microorganisms have been identified and applied to remove herbicides from natural environments. Several bacterial strains, such as *Paracoccus* sp. strain M-1 (Jia et al. 2006), *Sphingomonas* sp. strain DC-6 (Chen et al. 2016), *Ochrobactrum* sp. strain PP-2 (Zhang et al. 2019), *Paracoccus* sp. strain FLN-7 (Zhang et al. 2012), and *Comamonas* strain SWP-3 (Zhang et al. 2020a), have been reported to have the ability to degrade propanil. Microbial strains such as *Acinetobacter baumannii* DT (Duc et al. 2021), *Pseudomonas aeruginosa* JD115 (Luo et al. 2015), and *Bacillus altitudinis* A16 (Kaur and Goyal 2020) have likewise been reported to effectively degrade chloroacetamide herbicides, such as acetochlor. The enzymes reported to contribute to amide herbicide degradation in these bacteria are mainly amidohydrolases or amidases. For instance, two amidohydrolases (PuhA and PuhB) responsible for linuron hydrolysis have been identified from *Arthrobacter globiformis* D47 (Turnbull et al. 2001) and *Mycobacterium brisbanense* JK1 (Khurana et al. 2009), respectively. A novel amidase PsaA identified from *Bosea* sp. was able to convert the herbicide propanil to its metabolite (Zhang et al. 2023). Another novel amidase PamH with broad substrate spectra was identified from *Paracoccus* sp. M-1 and could degrade a variety of organophosphorus insecticides and amide herbicides, such as phosphamidon, dichlorvos, propanil, acetochlor, and pretilachlor (Shen et al. 2012). Although there is an abundance of bacterial species present in soil, studies have suggested that more than 99% of these bacteria cannot be cultured using traditional laboratory techniques (Pham and Kim 2012). This means that the vast majority of microorganisms in the soil environment remain unexplored, owing to the barriers of bringing microorganisms to in vitro cultivation. To this end, metagenomics, which has emerged as a new field of research developed over the past decade, has proved itself a remarkable technique which is culture-independent, thereby representing a powerful tool for discovering novel biocatalysts from unculturable microorganisms (Schloss and Handelsman 2005). Several novel enzymes with a broad range of applications have been screened from soil metagenomic libraries, such as esterase, protease, cellulase, and amylase (Xing et al. 2012). However, to date, few studies have been published on amidohydrolases or amidases that degrade amide herbicides derived from metagenomic libraries, mainly because of the absence of effective screening methods.

In the current study, a novel amidohydrolase gene (AmiH52) was identified and cloned from a soil metagenomic library. The recombinant enzyme expressed in *E. coil* BL21 was further purified and biochemically characterized in terms of temperature and pH. The effect of various metal ions, organic solvents, and detergents on the activity of the enzyme AmiH52 was also addressed. Meanwhile, the degradation effect of AmiH52 on different amide herbicides was described in detail. Finally, molecular modeling and docking were performed to preliminarily explain the degradation mechanism of herbicides by AmiH52.

## Materials and methods

### Chemical reagents

Diethyl terephthalate (DET) and several amide herbicides, including acetochlor, propanil, diuron, carbosulfan, and diflubenzuron, were procured from Shanghai Macklin Biochemical Co., Ltd. A group of *p*-nitrophenyl esters with different acyl chain lengths (C2-C16) was purchased from Shanghai Yuanye Biological Technology Co., Ltd. Other chemicals and reagents, such as methanol, dimethyl sulfoxide (DMSO), and isopropyl β-D-1-thiogalactopyranoside (IPTG), were obtained from Nanjing Soda Biological Technology Co., Ltd.

### Strains, plasmid, and culture conditions

*E. coli* competent cells (DH5α and BL21 (DE3)) were bought from Nanjing Vazyme Biotech Co., Ltd. Plasmids, including pET-28a and pUC118 vector, were obtained from Miaoling Biotechnology Co., Ltd. (Wuhan, China). Reaction enzymes (restriction endonucleases including *Sau3A*I, *Nco*I, *Hind*III, and T4 ligase) and DNA/protein markers were procured from Takara Bio (Kusatsu, Japan). All bacterial strains were grown in a commonly used Luria–Bertani (LB) broth at appropriate temperatures.

### Construction of the metagenomic library

The soil samples were collected from a farmland site in Dalian, Liaoning, China (Latitude—38.5445 N, longitude—121.3609 E). The entire environmental DNA was extracted based on a standard protocol (Brady 2007). The DNA fragments (~ 40 kb) were recovered from the agarose gel after electrophoresis and purified using the electroelution method. The purified DNA fragments were then ligated to the pWEB plasmid, which was further packaged using lambda phage and transfected into *E. coli* EPI100 for building a metagenomic library using the commercial Cosmid Cloning Kit (purchased from Epicentre Biotechnologies).

### Identification and isolation of positive clones

LB agar plate with 100 μg/mL ampicillin and 2.5 mM DET was used for function-driven screening of a soil metagenomic library (1.3 × 10^5^ clones). After the library clones were incubated at 37 ℃ for 24 h, colonies that showed clear halos around them on the agar plate were identified as positive clones and were picked for the next analysis. To further identify the specific hydrolase gene, the plasmids of positive clones were extracted using commercial kits and then partially digested by *Sau3A*I (Takara, Japan). The resulting 1–5-kb DNA fragments were recovered, subcloned into the pUC118 vector, and transformed into *E. coli* DH5α. Similarly, the positive subclone was identified according to DET-degrading activity on the LB agar plate and sequenced at Tsingke Biotech Co., Ltd. (Nanjing, China).

### Sequence analysis

Open reading frame (ORF) Finder searches were used for ORF identification at the NCBI online website. The amino acid sequence of a complete ORF was then subjected to the BlastP for homology searches. The phylogenetic tree of these aligned sequences was drawn using the MEGA-X software package based on the neighbor-joining method. The predicted molecular weight of a protein was determined by the ExPASy online tool (https://www.expasy.org/). The presence of signal peptide was predicted with the SignalP 5.0 server (https://services.healthtech.dtu.dk/services/SignalP-5.0/).

### Cloning

**Fig. 1:**
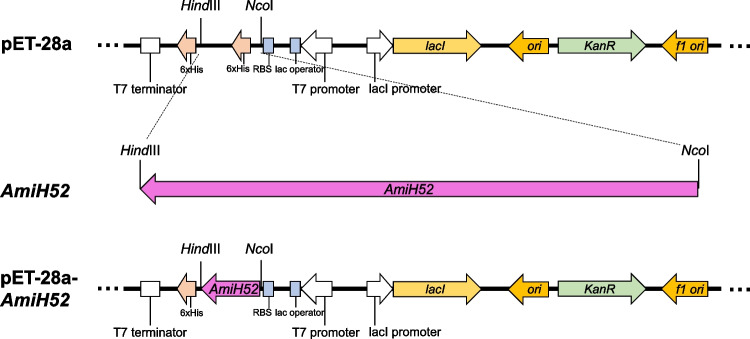
Physical map of recombinant AmiH52

A putative gene of AmiH52 was amplified by PCR using the following primers: *amih52*-F (5′-CATGCCATGGGCATGGGGCGAAGCGCGGAAG-3′) containing a *Nco*I recognition site shown in underlined, and *amih52*-R (5′-CCCAAGCTTAGTAAGGCCCAGCAACGCT-3′) containing a *Hind*III recognition site shown in underlined. Both the PCR product and pET-28a vector were digested with *Nco*I and *Hind*III. The digested DNA fragment was then ligated into a pET-28a using T4 ligase at 16 ℃ for 12 h. The physical map of the recombinant plasmid is shown in Fig. 1. The recombinant plasmid with a C-terminal six-histidine affinity tag was transformed into *E. coli* BL21 (DE3) competent cells for protein expression.

### Protein expression and purification

Bacteria harboring recombinant plasmid were grown at 37 ℃ and 180 rpm. When the cell culture reached an OD600 of 0.6–0.8, IPTG (0.5 mM) was added into the medium to induce the protein expression at 16 ℃ for 24 h. The cultures were then harvested by centrifugation (10,000 g, 4 ℃, 10 min) and re-suspended in 20 mL phosphate buffer saline (PBS) buffer (100 mM, pH 8.0) and disrupted by an ultrasonic homogenizer (SCIENTZ-IID, Ningbo, Zhejiang, China) using an ice bath for 30 min. Next, the recombinant proteins in the crude lysates were purified by Ni–NTA affinity chromatography. Enzyme purity and molecular mass were determined using sodium dodecyl sulfate–polyacrylamide gel electrophoresis (SDS-PAGE). Each protein concentration was measured three times on the NanoDrop 2000c spectrophotometer (Thermo, USA).

### Characterization of enzyme

A variety of *p*-nitrophenyl (*p*NP) esters having different acyl chain lengths (*p*NP acetate (C2), *p*NP butyrate (C4), *p*NP hexanoate (C6), *p*NP caprylate (C8), and *p*NP laurate (C12)) were chosen as substrates to investigate the substrate specificity of AmiH52. The reaction mixture consisted of 50 μL of *p*-nitrophenyl esters (10 mM), 50 μL of enzyme solution, and 400 μL of Tris–HCl buffer (100 mM, pH 8.0). The activity of the enzyme was determined based on the enzyme catalyzing the substrates to release *p*-nitrophenol, a chromogenic product whose absorbance can be measured at 405 nm. An international unit (U) for enzyme activity is defined as the amount of enzyme that produces 1 μmol of *p*-nitrophenol in 1 min.

The optimum temperature of AmiH52 was measured by mixing the appropriate amount of enzyme and substrate (1 mM *p*NP butyrate) in Tris–HCl buffer (100 mM, pH 8.0) at different temperatures (20–70 ℃). The thermostability of AmiH52 was investigated by pre-incubating the enzyme at 20–70 ℃ for 2 h. The residual activity was subsequently measured at its optimum temperature. To determine the optimum pH, AmiH52 was examined in different pH buffer solutions (pH 3.0 to 10.0) at 40 ℃ for 5 min. Subsequently, the stability of AmiH52 at these pH values was investigated by pre-incubating the enzyme with varied pH range buffers at 4 ℃ for 1 h. The effects of various chemical reagents on the AmiH52 activity were determined by adding various organic solvents (methanol, ethanol, isopropanol, n-butanol, acetone, DMSO, cyclohexane, and acetonitrile, at concentrations of 10% and 30%), metal ions (Na^+^, Mg^2+^, Ca^2+^, Mn^2+^, Al^3+^, Co^2+^, Fe^3+^, Cu^2+^, and Zn^2+^, each at 5 mM), and detergents (CTAB, Tween-80, Triton X-100, SDS, and EDTA, at 1% concentration) to the reaction solution at 40 ℃ for 5 min. The enzyme activities were determined according to the above procedure. In these enzymatic reaction solutions, the activity of the enzyme without any additive was used as a control and set at 100%.

### Degradation of amide herbicides

The purified AmiH52 was prepared and stored at 4 ℃ for the degradation assay. The enzyme activity against amide herbicides was determined in 400 μL Tris–HCl buffer (100 mM and pH 8.0) supplemented with 10 μg/mL herbicide substrate (acetochlor, propanil, diuron, carbosulfan, and diflubenzuron) and 200 μL purified enzyme at 40 ℃ for 1 h, 2 h, and 8 h, respectively. A reaction solution without enzyme addition was used as the control group. Three replicates were set up for each group. After the reaction, the enzyme was inactivated by heating for 10 min in a boiling water bath. In each experimental group, 200 μL of supernatant was collected from each vial and passed through a sterile 0.22-μm filter membrane to obtain the final measurement samples, which were then placed in a 4 ℃ refrigerator for temporary storage.

### Product analysis

To evaluate the degradation efficiency for each amide herbicide, an LC-20A high-performance liquid chromatography (HPLC) system (Shimadzu, Japan) coupled with a photodiode array detector and an Agilent ZORBAX SB-C18 column (4.6 × 150 mm, 5 µm) was employed to separate compounds in the reaction mixtures. The mobile phase was phase A (pure water) to phase B (acetonitrile) (20:80, v/v), and the flow rate was constant at 0.8 mL/min. The injection volume was 20 µL, and the column temperature was set to 25 ℃.

To further analyze the degradation products, gas chromatography-mass spectrometry (GC–MS) (Thermo Scientific TSQ 9000, USA) was harnessed to determine the individual substances within a sample. The enzyme reaction solutions in the tube were dried using nitrogen for 30 min. Subsequently, 200 μL of HPLC-grade acetonitrile was added to the tube, and the product was dissolved and dispersed in the acetonitrile by vortex shaking for a short time. The samples were passed through a 0.2-μm filter for the next GC–MS analysis. The GC–MS conditions were as follows: the column was an HP-5 column (0.25 μm × 0.25 mm × 30 m); a flow rate was set to 1 mL/min; the injection method was splitless injection GC; the injection port temperature and ion source temperature was 280 and 250 ℃, respectively. The ramp-up procedure of the GC column temperature chamber was as follows: hold at 60 ℃ for 3 min, increase to 200 ℃ at a rate of 15 ℃/min, then increase to 280 ℃ at a rate of 10 ℃/min, and hold at this temperature for 5 min.

### Molecular docking

The small molecule’s three-dimensional (3D) structure was obtained from PubChem online database. The 3D structure of AmiH52 was predicted at I-TASSER online website (https://seq2fun.dcmb.med.umich.edu//I-TASSER/). Molecular docking was conducted using the AutoDock Vina program, and PyMoL software was harnessed for 3D visualization of protein complex and analysis of the results in protein structure: the interaction between AmiH52 and amide herbicide.

### Molecular dynamics (MD) simulation

Gromacs 2022.3 software was used for molecular dynamics simulation. For small-molecule preprocessing, AmberTools22 was used to add the General AMBER Force Field (GAFF) to small molecules, while Gaussian 16W was used to hydrogenate small molecules and calculate RESP potential. Potential data will be added to the topology file of the molecular dynamics system. The simulation conditions were performed at a static temperature of 300 K and atmospheric pressure (1 bar). Amber99sb-ildn was employed as a force field, water molecules were used as the solvent (Tip3p water model), and the total charge of the simulation system was neutralized by adding an appropriate number of Na^+^ ions. The simulation system adopts the steepest descent method to minimize the energy and then carries out the isothermal isovolumic ensemble (NVT) equilibrium and isothermal isobaric ensemble (NPT) equilibrium for 100,000 steps, respectively, with the coupling constant of 0.1 ps and the duration of 100 ps. Finally, the free molecular dynamics simulation was carried out. The process consisted of 5,000,000 steps, the step length was 2 fs, and the total duration was 100 ns. After the simulation was completed, the built-in tool of the software was used to analyze the MD trajectory and calculate the root mean square variance (RMSD) of ligand, protein, and complex; root-mean-square fluctuation (RMSF) of AmiH52 residues; the number of hydrogen bonds; and solvent-accessible surface area (SASA) of proteins.

## Results

### A novel amidohydrolase

**Fig. 2:**
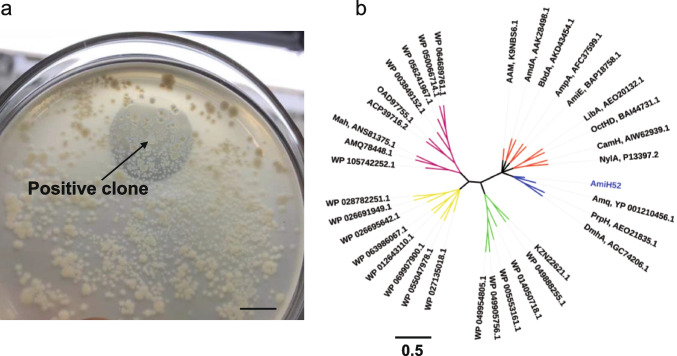
A novel amidohydrolase AmiH52 identified from the metagenomic library. **a** A positive clone formed a clear halo on the DET-containing LB agar plate. Scale bar, 1 cm. **b** Phylogenetic analysis of the newly identified AmiH52. The biochemically characterized amidohydrolases or amidases (available from the NCBI nonredundant protein sequence database and the Swiss-Prot database) were aligned with AmiH52 (blue). The bar represents a 0.5 amino acid difference per site

After the screening of the soil metagenomic library, a positive clone on the DET-containing agar plate was identified by the presence of a clear halo (Fig. 2a). Further, a sub-cloning library was constructed to obtain the specific hydrolase gene. Similarly, a positive sub-clone was identified and sequenced. The gene sequence of sub-clone SC52 was input into the ORF Finder, showing that there were 29 ORFs, of which ORF17, with a length of 984 bp and consisting of 328 amino acid residues, was the most likely functional motif. The encoding protein was named AmiH52. The amino acid sequence was then subjected to NCBI-BLASTp, and it was found that the homologous proteins of AmiH52 belonged to the amidohydrolase superfamily. It showed the highest similarity of 73.09% with the amidohydrolase (MBK9945108.1) from *Kouleothrix* sp. and 71.43% similarity with the amidohydrolase superfamily protein (WP_141612206.1) from *Litorilinea aerophila*. Furthermore, the evolutionary relationship between AmiH52 and its homologous amidohydrolases from varied hosts was analyzed (Fig. 2b). It revealed that AmiH52 was clustered into a clade with amidohydrolase DmhA (AGC74206) from *Sphingomonas* sp. DC-6 (Chen et al. 2016), histone-like hydrolase PrpH (AEO21835) from *Sphingomonas* sp. Y57 (Zhang et al. 2011), and α/β hydrolase Amq (YP_001210456) from *Arthrobacter nitroguajacolicus* Rü61a (Kolkenbrock et al. 2007). However, the similarities between AmiH52 and these functional hydrolases obtained from the NCBI/Swiss-Prot database were quite low (less than 15%) after performing the multiple sequence alignment by CLUSTALW. Therefore, based on sequence analysis and phylogenetic relationship, AmiH52 was judged as a novel amidohydrolase.

ExPASy program analysis revealed that the theoretical molecular mass of AmiH52 was 37.2 kDa, and the isoelectric point was 6.63. SignalP-5.0 analysis suggested that the signal peptide score of AmiH52 was extremely low, implying there is no signal peptide for the AmiH52 sequence. TMHMM-2.0 online server tool showed that there were no transmembrane helices, and the protein sequences of AmiH52 are all located inside the cell membrane; thus, the heterologously expressed protein in *E. coli* will be released using ultrasonication treatment.

### Protein expression

**Fig. 3:**
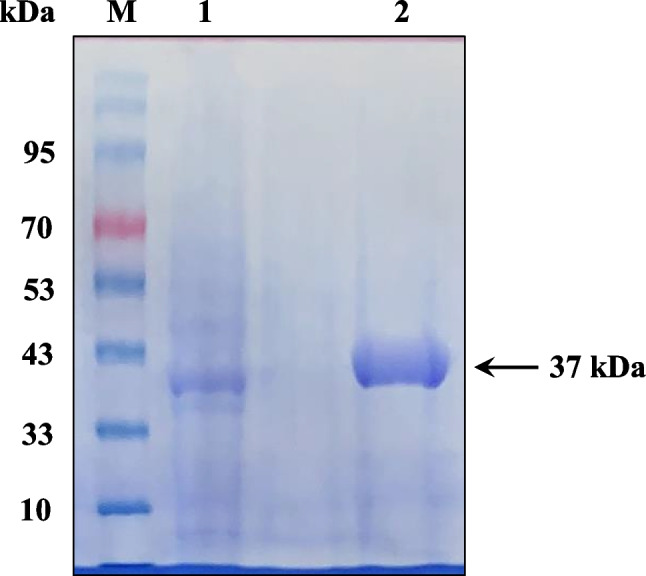
SDS-PAGE of purified protein. 1: crude enzyme; 2: purified AmiH52; M: standard protein molecular weight marker

The amide hydrolase AmiH52 was successfully overexpressed in *E. coli* BL21 (DE3). The protein purity was confirmed by using SDS-PAGE. Figure 3 shows that the protein band on the gel is single and clear, indicating that the recombinant protein is pure. Moreover, the recombinant enzyme has a molecular weight of approximately 37 kDa, which is in agreement with the predicted molecular weight of 37.2 kDa.

### Characterization of AmiH52

**Fig. 4:**
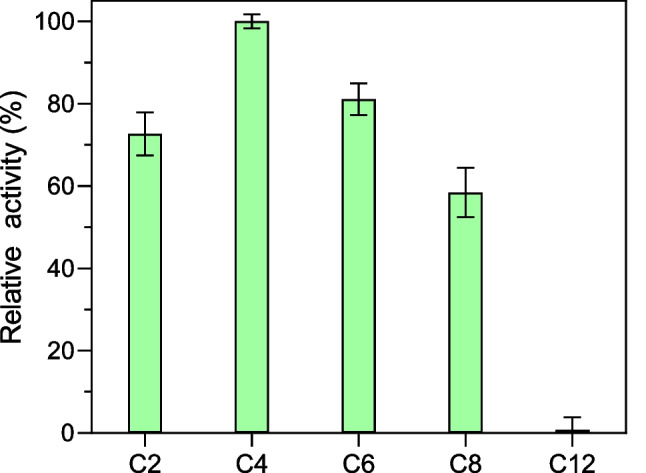
Substrate preference toward *p*-nitrophenyl esters with various acyl chain lengths. C2: *p*NP acetate, C4: *p*NP butyrate, C6: *p*NP hexanoate, C8: *p*NP caprylate, C12: *p*NP laurate

Given that AmiH52 showed high hydrolytic activity against synthetic ester DET, *p*-nitrophenyl esters with various lengths of acyl chains were selected to determine the substrate specificity. The recombinant enzyme AmiH52 exhibited the highest hydrolysis activity toward *p*NP butyrate (C4) (Fig. 4), and the specific enzyme activity reached 186.83 U (2669 U/mg). The hydrolysis activity of *p*NP esters with side chain length of C2-C8 was generally high; however, the catalytic activity of *p*NP esters with long side chain (C12) decreased sharply, indicating that the substrate specificity of AmiH52 is broad, and the preference object is esters with short acyl chains rather than esters with long-chain fatty chains, a typical esterase activity.

**Fig. 5:**
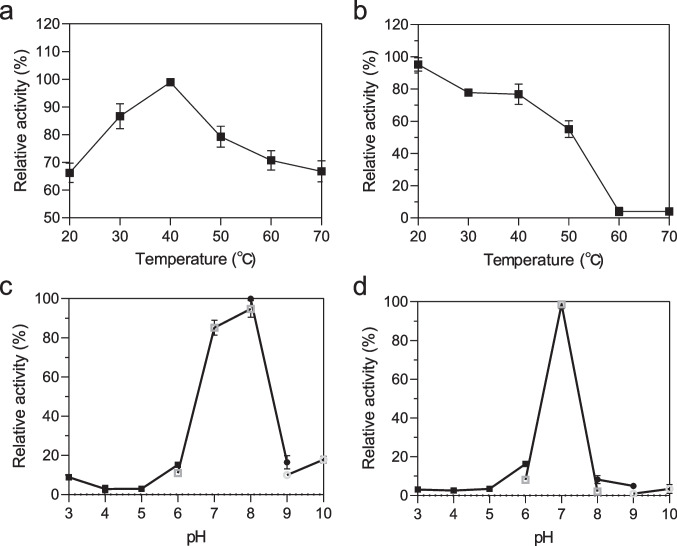
Characterization of AmiH52. Effects of temperature (**a**) and pH (**c**) on the enzyme activity of AmiH52. Determination of thermal (**b**) and pH (**d**) stability of the recombinant enzyme

Furthermore, the recombinant enzyme AmiH52 exhibited the maximum activity at 40 ℃ (Fig. 5a). In the range of 20–70 ℃, the relative enzyme activity was generally higher than 60%. Figure 5b shows that the activity of the enzyme displayed a downward trend with increasing temperature. In the water bath temperature ranging from 20 to 50 ℃, the residual activity of AmiH52 is above 50%, while it decreased sharply when the temperature rises from 50 ℃. For the impact of pH on enzyme activity, AmiH52 exhibited the maximum activity at pH 8.0 (Fig. 5c). The results of the pH stability experiment showed that the AmiH52 displayed relatively high stability in the pH range of 6–8. However, it has less than 10% enzyme activity when the pH is higher than 9.0 and lower than 5.0 (Fig. 5d).

**Fig. 6:**
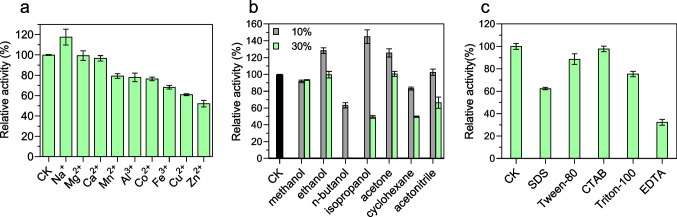
The influence of different metal ions (**a**), organic solvents (**b**), and detergents (**c**) on the enzymatic activity of AmiH52

Next, the influence of different metal ions, organic solvents, and detergents on the enzyme activity of AmiH52 was determined. For metal ions, Na^+^ could slightly enhance the hydrolysis ability of the recombinant enzyme and increase its enzyme activity by 20% (Fig. 6a) when compared to the control group. Mg^2+^ and Ca^2+^ had no obvious impact on the enzyme activity of AmiH52. Mn^2+^, Al^3+^, Co^2+^, Fe^3+^, Cu^2+^, and Zn^2+^ inhibited the enzyme activity more and more strongly, but the overall enzyme activity remained above 50%, indicating that AmiH52 had a high tolerance to different metal ions. The effect of different organic solvents on enzyme activity is shown in Fig. 6b. At a low concentration of 10%, ethanol, isopropanol, and acetone could significantly increase the catalytic activity of the AmiH52 by more than 20%. For methanol and acetonitrile, there is little effect on the enzymatic activity. The catalytic activity of the AmiH52 was slightly inhibited by n-butanol and cyclohexane, with the activity reduced by 20 to 40%. When the chemicals were supplied at a high concentration of 30%, no apparent effect of methanol, ethanol, and acetone on the activity of the enzyme was observed, while the other organic reagents significantly reduced the enzyme activity. Among them, n-butanol almost completely inhibited the enzyme activity of the AmiH52. For the effect of different detergents on AmiH52 (Fig. 6c), it was found that EDTA reduced the enzyme activity to 30%, while SDS and Triton X-100 reduced the activity to 60% and 80%, respectively. Tween-80 and CTAB had a weak inhibitory effect on AmiH52, and the relative activity of the enzyme remained above 90%.

### Amide herbicide degradation

**Fig. 7:**
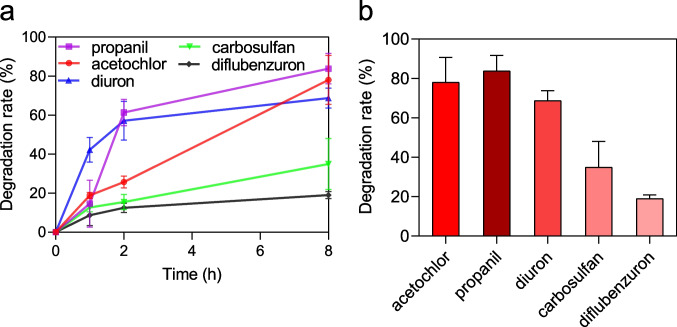
Amide herbicide degradation. **a** The degradation rate with the time of the five amide herbicides. **b** The degradation rate at 8 h of the five amide herbicides

The degradation of five herbicides (acetochlor, propanil, diuron, carbosulfan, and diflubenzuron) by amidohydrolase AmiH52 with time was studied. As shown in Fig. 7a, the degradation rate of propanil was fast at the initial stage, reaching 60% at 2 h and the highest degradation rate of 84% at 8 h (Fig. 7b). The degradation rate of diuron reached 57% at 2 h and 69% at 8 h (Fig. 7b). Degradation reaction rate of acetochlor was slow at the initial stage of degradation, only 20% at 2 h, but reached 78% at 8 h (Fig. 7b). The degradation rate of carbosulfan and diflubenzuron at 8 h was below 30%.

**Fig. 8:**
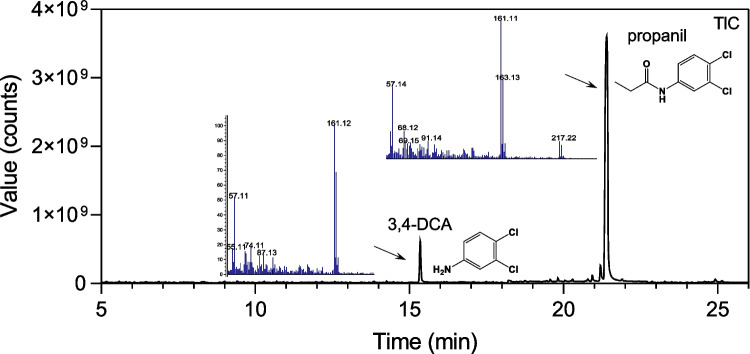
Degradation pathway analysis of propanil using GC–MS

Given that AmiH52 exhibited the highest degradation activity toward propanil, it was selected for GC–MS analysis. Figure 8 shows that two compounds, including propanil and its product, were identified at the retention time of 21.35 min and 15.45 min, respectively. Based on the mass spectra and library hit, it was found that AmiH52 is able to break the amide bond of propanil to produce 3,4-dichloroaniline (3,4-DCA).

### Docking study

**Fig. 9:**
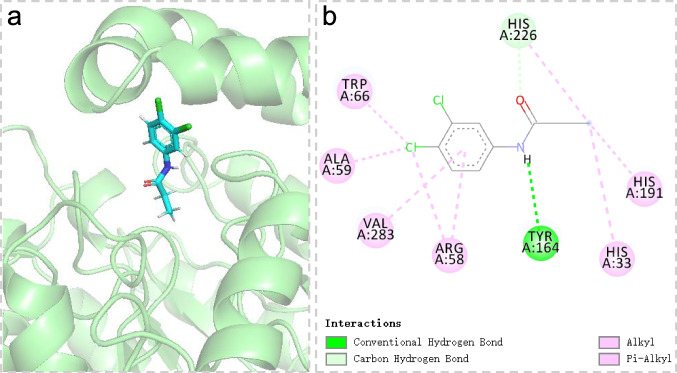
Interactions between AmiH52 and propanil. (**a**) The propanil was docked into the active pocket site of AmiH52; (**b**) Several amino acid residues were involved in the interaction with propanil. Those interactions included conventional hydrogel bond, carbond hydrogen bond,alkyl-alkyl interactions and pi-alkyl interactions

The molecular docking method was employed to understand the specific binding modes of propanil in contact with AmiH52. The propanil was docked into the active pocket site of AmiH52 (Fig. 9a). Several amino acid residues were involved in the interaction with propanil (Fig. 9b). In detail, Tyr164 is able to form a conventional hydrogen bond with the N–H of propanil, which is beneficial for the stabilization of the protein complex during the catalytic process. Trp66, Ala59, Val283, Arg58, His33, and His191 can form pi-alkyl/alkyl interactions with propanil. His226 is responsible for the formation of carbon-hydrogen bonds with the amide group of propanil. The novel AmiH52 represented an atypical amidohydrolase, with low sequence similarity to its close homologous protein Amq (YP_001210456.1) containing a typical catalytic triad of S155, E235, and H266. It was hypothesized that His226, as one of the catalytic triads of AmiH52, played an important role in the catalytic hydrolysis of propanil. The catalytic His226 first acted as a base to deprotonate the nucleophile (possibly serine or threonine) to attack the amide bond of propanil in the primary step, which will generate an enzyme intermediate. Then, His226 again acted as a base to deprotonate the water molecule so that it could break the carbonyl group of the intermediate complex to release the product 3,4-DCA. The free catalytic triads will be ready to attack the next substrate molecules.

### MD simulation

**Fig. 10:**
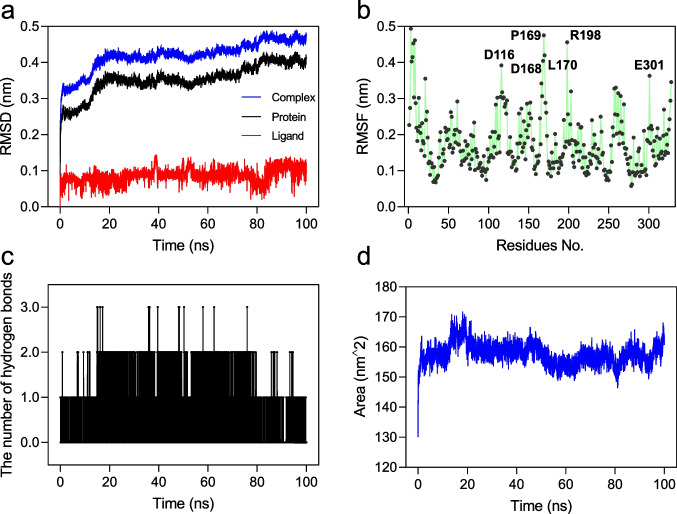
MD simulation to study the binding stability of propanil in complex with AmiH52. **a** RMSD values of ligand (red line), protein (black line), and complex (AmiH52 and propanil) (blue line) calculated over 100 ns. **b** RMSF result of 328 residues in AmiH52. Several residues (D116, D168, P169, L170, R198, and E301) with obvious fluctuation are marked. **c** The number of hydrogen bonds formed between AmiH52 and propanil changed with time. **d** SASA analysis of AmiH52 during the MD simulation process

The binding stability of propanil in complex with AmiH52 was further analyzed based on the 100-ns MD simulation (see the video in SI). Values of the root mean square deviation (RMSD) and root mean square fluctuation (RMSF) were calculated to determine the overall discrepancy of the protein molecule and the individual residue fluctuation during the MD simulation, respectively. The results showed that the RMSD values of the complex and protein increased rapidly during the first 20 ns of the simulation and then gradually leveled off (Fig. 10a). Overall, the RMSD values of all molecules varied within a small range, indicating that the binding between AmiH52 and propanil was stable. The RMSF values of the residues bound to propanil were generally small, while some residues such as D116, D168, P169, L170, R198, and E301 in the nonbound parts exhibited large RMSF values (Fig. 10b), indicating that the binding of propanil has a certain impact on the stability of AmiH52. Moreover, the hydrogen bond numbers between AmiH52 and propanil were calculated (Fig. 10c). The results showed that many hydrogen bonds (numbering from 0 to 3) were formed between protein and small molecules during the whole simulation process, suggesting hydrogen bonds are the key interactions between residues in the protein and some important groups located in the small molecules. Additionally, solvent-accessible surface area (SASA) was determined between proteins and propanil molecules (Fig. 10d). It showed that the SASA value of the protein was large before the protein was combined with the small molecule, but slightly decreased after the combination, indicating that the combination of small molecules was able to reduce the surface area of the protein.

## Discussion

Amide herbicide is one of the most extensively used pesticides in the world. Owing to their low degradation rate, chemical stability, and highly water-soluble properties, however, residual amide herbicides gradually accumulate in the soil and water environment, posing a serious threat to the ecosystem (Han et al. 2022; Jolodar et al. 2021). Compared to the physical and chemical methods, biodegradation has been identified as a powerful, green, sustainable, and economical strategy to reduce amide herbicide pollution (Huang et al. 2017). In recent years, several microorganisms with herbicide-degrading capabilities have been identified and isolated from various environmental samples, especially from soil. However, those strains used for herbicide degradation, which were isolated based on traditional laboratory techniques, only account for less than 1% of microbes in the environmental samples. Metagenomics has already opened new avenues of research by providing access to far more microbial diversity in environmental contexts than traditional culturable manners (Handelsman 2004; Rashid and Stingl 2015). Function-driven screening is the preferred method because it is more straightforward, efficient, targeted, and more likely to enable the discovery of new genes with new functions when compared to sequence-based screening (Ngara and Zhang 2018). Selecting an appropriate substrate for function/activity screening is a major consideration. For example, tributyrin is frequently used for screening esterase (Nazarian and Arab 2022; Popovic et al. 2015), while carboxymethyl cellulose is often used for cellulase screening (Islam and Roy 2018). The corresponding clear halos or the color change on the substrate-containing plate has demonstrated that positive clones carry putative hydrolase genes. However, for amide herbicide screening, the positive clones cannot either form clear halos surrounding the colony or produce color change due to their highly water-soluble and structurally stable properties. Hence, the effective screening method for amide herbicide from metagenomic libraries has yet to be fully developed.

In the current study, diethyl terephthalate (DET) was used as a functional screening substrate for amide herbicide-degrading enzymes. DET is the most widely known synthetic ester compound due to its excellent material properties and has been used for the screening of polyethylene terephthalate (PET)–degrading strains (Liu et al. 2018). Interestingly, a novel amidohydrolase AmiH52 (the highest similarity of 73.09% with the amidohydrolase (MBK9945108.1) from *Kouleothrix* sp.) with strong ester hydrolysis and amide herbicide–degrading activities was identified. This ability is called enzyme promiscuity, playing a crucial role in ancestral strains for adaptation to evolutionary pressure or changing environments (Leveson-Gower et al. 2019). Many enzymes have been identified as promiscuous enzymes including carboxylesterases EstCE1 from a soil metagenome (Elend et al. 2006) and MsAcT from *Mycobacterium smegmatis* (Godehard et al. 2020), serine hydrolase (CalB) from *Candida antarctica* (Svedendahl et al. 2005), and C5 methyltransferase isolated from *Haemophilus influenza* (Cohen et al. 2002). The promiscuous AmiH52, presented in this study, is possibly the first example of amidohydrolase with high esterase activity (2669 U/mg for *p*NP ester C4). Moreover, it is suggested that we can use some certain ester compounds like DET to screen potential amidohydrolases from the metagenomic libraries.

High-level expression of recombinant proteins in *E. coli* often leads to the aggregation of the expressed protein and the formation of the inclusion body. Previous studies showed that heterologous expression of amidohydrolase LahB (Zhang et al. 2020b) and amidase Mah (Zhang et al. 2019) in *E. coli* BL21(DE3) has not been successful mainly because the inclusion bodies formed in the host. To this end, a number of strategies have been applied to increase protein solubility, such as lowing the induction temperature, reducing the inducer concentration and induction time, and promoter replacement (Gutierrez-Gonzalez et al. 2019; Muhlmann et al. 2017). In this study, the expression of recombinant enzyme AmiH 52 was induced by IPTG (0.5 mM) at 16 ℃ for 24 h and successfully overexpressed in *E. coli* BL21(DE3), thus providing a reference condition for amidohydrolase expression that can be used in future similar studies.

Among various amide herbicides, propanil is produced globally and is ranked among the top 20 pesticides used in agriculture in the USA (Roberts et al. 2009). Until now, a number of amidase/amidohydrolase which contribute to propanil degradation have been identified from a range of microorganisms. For instance, amidase/amidohydrolase AmpA and PamH were obtained from *Paracoccus* strain, and PrpH and DmhA were isolated from the *Sphingomonas* genus (Zhang et al. 2019). Most of these enzymes belong to amidases with extremely different substrate spectrums. For example, AmpA could efficiently hydrolyze propanil, dimethoate, chlorpropham, and omethoate, with a Km value of 29.5 µM for propanil (Zhang et al. 2012). PamH showed high activity against propanil, benzeneacetamid, propanamide, benzamide, pyrazinamide, and acetamide. Propenamide is the most suitable substrate for PamH (Shen et al. 2012). AmiH52 also exhibited a broad range of degrading activity against five amide herbicides, including acetochlor, propanil, diuron, carbosulfan, and diflubenzuron. It showed the strongest degradability toward propanil. The degradation rate of propanil reached 60% at 2 h and the highest degradation rate of 84% at 8 h. Furthermore, the degradation product of propanil by AmiH52 was determined as 3,4-dichloroaniline (3,4-DCA), which is consistent with previous studies (Herrera-Gonzalez et al. 2013; Zhang et al. 2011, 2019). In contrast, a mixed culture of four bacterial strains, which were isolated from the sediment slurry, transformed 90% of propanil within 10 days in liquid media (Oanh and Duc 2021). Another study reported that 81% of propanil was degraded after 100 days of incubation in an anaerobic soil environment (Pettigrew et al. 1985). Rafael et al. showed that an *Enterobacter cloacae* strain obtained from rice field soil was able to eliminate 100% of propanil in 28 days (Roehrs et al. 2012). Consequently, it is obvious that this study demonstrates the huge potential of biocatalyst AmiH52 identified from the metagenome and provides a strong and promising candidate for the remediation of a propanil-polluted environment.

## Conclusion

In the present study, a novel amidohydrolase gene was identified and isolated from a soil metagenomic library, which was heterologously expressed in *E. coli* BL21(DE3). The recombinant enzyme AmiH52 exhibited the highest activity at 40 ℃ and pH 8.0. AmiH52 exhibited both esterase and amidohydrolase activities. It showed the highest hydrolysis activity toward *p*NP butyrate (C4) (2669 U/mg). Several amide herbicides can be degraded by AmiH52. In particular, it displayed the strongest activity against propanil, with the highest degradation rate of 84% at 8 h. GC–MS analysis suggested that propanil was transformed into 3,4-DCA. The molecular docking study revealed that several key amino acid residues, such as Tyr164, Trp66, Ala59, Val283, Arg58, His33, His191, and His226, are involved in the interactions with propanil. Our MD simulations have revealed that the binding between AmiH52 and propanil was stable. In summary, this study has provided a function-driven screening method for amide herbicide hydrolase from the metagenomic libraries and a promising propanil-degrading amidohydrolase AmiH52 for potential environmental applications.

### Supplementary Information

Below is the link to the electronic supplementary material.

Supplementary file1 (DOCX 19 KB)

Supplementary file2 (MP4 2770 KB)

## Data Availability

All the data is included in the manuscript. The raw data is available upon a reasonable request.
