# Supplementary material for: Characterization of a novel amidohydrolase with promiscuous esterase activity from a soil metagenomic library and its application in degradation of amide herbicides
Source: Environ Sci Pollut Res Int. 2024 Feb 21;31(14):20970–82. doi: 10.1007/s11356-024-32362-6 (PMC10948491; doi:10.1007/s11356-024-32362-6)
Supplement: Supplementary file 1 — Supplementary file1 (DOCX 19 KB) [file 11356_2024_32362_MOESM1_ESM.docx]

**Characterization of a novel amidohydrolase with promiscuous esterase activity from a soil metagenomic library and its application in degradation of amide herbicides**

Shengwei Sun^1,2^, Wanqi Chen^1^, Kailin Peng^1^, Xueyingzi Chen^1^, Jinju Chen^2,3*^

^1^ Key Laboratory of Food Processing and Quality Control, College of Food Science and Technology, Nanjing Agricultural University, Nanjing 210095, PR China

^2^ School of Engineering, Newcastle University, Newcastle upon Tyne NE1 7RU, UK

^3^ Department of Materials, Loughborough University, LE11 3TU, UK

^*^Corresponding author.

Postal address: Department of Materials, Loughborough University, LE11 3TU, UK

E-mail: [jinju.chen82@gmail.com](mailto:jinju.chen82@gmail.com) ; [j.chen4@lboro.ac.uk](mailto:j.chen4@lboro.ac.uk);

**Supplementary materials for gene and protein sequences of AmiH52:**

>gene sequence of AmiH52

ATGGGGCGAAGCGCGGAAGGACATGGTACGTTCATTCGTCCTTCGCCCCACCGTACCCGCCAGAGAGGCAGCAACAGCCAGATGATTGTCGATATCCACACACACACGCCTCAGTTCAAAGAAGCCGTACCGTCCGACAAGGTTGTGATGAACGACAAATGGCGACCGGATCGTGCGGTGCGCGCCACCAATAGTTGGGCCGACTATTTCGAGGCGATGAAGCCTGCCGTCAAGTCAGTCGTCTTTAATATCGCGTGGCATCCAGGCCACGGACATGACCGGTTAGCGGGGGGGGAAAGCACCGACACGAGTTGGTATGCCAGTGAAGGGCGCGGCAACTACAACGATGCGACCGCCACCTTTGTGCGCGCCCACCCCGACAAGCTCATCGGCTTTATGGCGCTACATCCCTTTGATGCCGACATGCTTGAGGAATTTGAGCGTTGTCGCACAGATTTAGGCATGAAAGGCGTCAAGCTCGGCGCCAATTATCAAATCTTCGACCCGCTTGAGACGCGTGCGCTTGCCATTTATGAAGCTGCACAAAAGCATGGTCTGCCTGTCCTCTTTCACCAAGGCACCTCCCCTGTCCGCACCGCGCCCATACGGTATGCCCACCCCCTCTTGACCGACGAAATCGCCATCCGCTATCCCGATCTCAAGATCATCATGGCGCATCTTGGTCATCCTTGGCAGGTGGACACCGCAGTCGTTGTCCGCAAACATCCCAATGTCTATGCGGACATGTCAGCCAACTTTTACCGCCCCTACTCGTTTTGGGAGCAGATGGTCAAGGCGATGGAATGGAACATCACCGACAAAATCCTATTCGGTACAGACTTCCCCGTCACCCACATGCAAGAATCCATCGACGCTTTCCGCAATATCAACAACATTGTCGAAGGCACAAAGTTGCCGCGCATCCCAGAGGAGGTAATCGAAGGAATTATTCACCGTGATTCGTTAGCGTTGCTGGGCCTTACTTAG

>amino acid sequence of AmiH52 (328)

MGRSAEGHGTFIRPSPHRTRQRGSNSQMIVDIHTHTPQFKEAVPSDKVVMNDKWRPDRAVRATNSWADYFEAMKPAVKSVVFNIAWHPGHGHDRLAGGESTDTSWYASEGRGNYNDATATFVRAHPDKLIGFMALHPFDADMLEEFERCRTDLGMKGVKLGANYQIFDPLETRALAIYEAAQKHGLPVLFHQGTSPVRTAPIRYAHPLLTDEIAIRYPDLKIIMAHLGHPWQVDTAVVVRKHPNVYADMSANFYRPYSFWEQMVKAMEWNITDKILFGTDFPVTHMQESIDAFRNINNIVEGTKLPRIPEEVIEGIIHRDSLALLGLT

**Supplementary trajectory video**

Video for the binding dynamics of propanil in complex with AmiH52 during MD simulations.
